# Nitric oxide role in anxiety-like behavior, memory and cognitive impairments in animal model of chronic migraine

**DOI:** 10.1016/j.heliyon.2020.e05654

**Published:** 2020-12-07

**Authors:** Parichehr Taheri, Fatemeh Mohammadi, Masoud Nazeri, Mohammad Reza Zarei, Goli Chamani, Mohsen Abedini Esfahlani, Farahnaz Taheri, Mohammad Shabani

**Affiliations:** a Department of Oral Medicine and Orofacial Pain, Kerman School of Dentistry, Kerman Oral and Dental Diseases Research Center, Kerman, Iran; b Intracellular Recording Lab, Kerman Neuroscience Research Center, Neuropharmacology Institute, Kerman University of Medical Sciences, Kerman, Iran

**Keywords:** Nitric oxide, Migraine, Cognitive function, Anxiety, Neuroscience, Cell biology, Physiology, Neurology, Psychiatry, Psychology

## Abstract

The occurrence of cognitive dysfunctions and anxiety and mood disorders has been shown to be higher in migraine patients. Nitric Oxide (NO) is a significant neurotransmitter in the pathophysiology of migraine, anxiety and neurodegenerative disorders. Therefore, the present study was conducted to evaluate the role of NO system in migraine-induced memory impairment and anxiety like behaviors. Nitroglycerin (NTG) was administered to the animals as an animal model of migraine and pretreatment with L-Arginine, L-NAME and saline were implemented to evaluate the role of NO system in possible cognitive impairments in animal model of migraine. Avoidance learning and memory performance, object recognition memory, anxiety-like behavior and motor activity were assessed using a shuttle box apparatus, novel object recognition, elevated plus-maze, and open field tests respectively. The data showed that the injection of nitroglycerin disturbs learning and memory and elicit anxiety like behavior in the animals. L-NAME administration suppressed the observed effect of nitroglycerin on memory and anxiety. Overall, the results indicated that nitric oxide system is implicated in memory impairments and anxiety like behavior in an animal model of migraine.

## 1 Introduction

Headache is a common neurologic disorder in the general population and approximately 90% of all headaches are primary headaches. Migraine is one of the most prevalent primary headaches associated with symptoms such as nausea, vomiting, photophobia, and phonophobia and a 5% prevalence in the general population and a significant burden for both the individual and society [1]. Migraine that undergoes progression clinically evolves to chronic migraine with minimum duration of four hours per day and occurrence in 15 or more days per month [1].

Although migraine mechanisms have not yet been totally clarified, it has been documented that trigeminal nerve fibers surrounding meningeal vessels is involved in migraine pathophysiology [2]. The activation and sensitization of trigeminovascular pathway leads to nociceptors activation and promote neurogenic inflammation by releasing vasoactive peptides such as nitric oxide (NO) and calcitonin gene related peptide (CGRP) [3].

NO is generated from the amino acid L-Arginine by nitric oxide synthase (NOS) in the body. NO is known as an intercellular signaling molecule with various neuromodulatory actions in the central nervous system. NO has been recognized to play an important role in many behavioral, cognitive and emotional processes and perception of pain [4]. The involvement of NO in learning and memory, synaptic plasticity, long-term potentiation (LTP) and the consolidation of long-term memory is demonstrated before [5]. In addition, it has been demonstrated that NO regulates activity of HPA axis and it has been implicated in regulation of anxiety and stress responses [6].

NO is an important neurotransmitter in pain pathways and it is considered to play a fundamental role in migraine pathophysiology [7]. It has been demonstrated that the administration of nitroglycerin (NTG) as a nitric oxide donor induces a headache in healthy subjects and more severe delayed headache in migraineurs [8].

A lower cognitive performance has been reported in painful conditions [9]. Numerous studies have addressed patients with migraine have worse cognitive performance compared to non-migraineurs, even outside headache attacks [10]. In particular, it has been demonstrated that migraine patients exhibit impaired visual and verbal memory, reduced information processing speed, executive dysfunction, and attention deficit [11].

Association between migraine headaches and psychiatric disorders has been well demonstrated in the literature [12]. Clinical and epidemiological studies indicated that this relationship is bidirectional, in that a significant proportion of migraineurs suffer from mood and anxiety disorders and vice versa [13]. It has been reported that anxiety disorders are two to ten times more prevalent in patients with chronic migraine than nonmigraineurs [14].

Using rodent pain models, it previously demonstrated that NO exerts a modulatory effect on pain-induced changes in anxiety [15] and learning and memory [5, 16]. Furthermore, it has been shown that alterations in NO synthesis mediate pain perception. However, the modulating effect of NO in migraine, as well as in anxiety, motor function and passive avoidance learning in this disease are not elucidated yet.

Since NO plays an important role in pain [17] and participates in release of migraine-related factors, including substance P and prostaglandin E2 [18] which are involved in pain [19, 20], the aim of this study was to study the role of NO system in migraine-induced alterations in anxiety-like behaviors, motor function and passive avoidance learning and memory.

Results of the current study might provide a basis for further research on the mechanisms and pathophysiology of cognitive impairments and anxiety in chronic migraine pain.

## 2 Materials and methods

### 2.1 Animals

Adult male Wistar rats weighing 230–270 g (n = 48) were used in this study. The animals were caged under a 12 h light/dark cycle in controlled condition with temperature of 22 ± 2 °C. Food and water were available ad libitum. Experimental protocols and procedures were approved by the Animal Research Ethics Committee of the Kerman Neuroscience Research Center (KNRC/EC/95-61). Animals were randomly divided into 6 groups (n = 8 rats for each group):

1 Saline-treated group (saline), rats received intraperitonealy saline for 9 days on alternate day (injections being made with 48h delays)
2 L-Arginine-treated group (L-Arginine), rats received intraperitonealy L-Arginine for 9 days (on alternate days, 10 mg/kg)
3 L-NAME-treated group (L-NAME), rats received intraperitonealy L-NAME for 9 days (on alternate days, 10 mg/kg)
4 NTG-treated group (NTG), rats received intraperitonealy NTG for 9 days on alternate day (injection was made every 48 h, 10 mg/kg)
5 NTG plus L-Arginine-treated group (NTG + L-Arginine), nitroglycerin-induced migraine rats received L-Arginine (10 mg/kg, i.p.) 1h before NTG administration for 9 days (on alternate days)
6 NTG plus L-NAME-treated group (NTG + L-NAME), nitroglycerin-induced migraine rats received L-NAME (10 mg/kg, i.p.) 1h before NTG administration for 9 days (on alternate days)

### 2.2 Migraine model establishment and experimental design

Experimental model of migraine was implemented by intermittent administration of 10 mg/kg NTG for 9 days (on alternate days) and has demonstrated reliable validity for the induction of migraine like pain in the animals in previous studies [21].

### 2.3 Behavioral assessment

Each group went through four different behavioral studies with 30 min intervals among each assay in the following order: anxiety like behavior assessment, open field test, novel object recognition and passive avoidance task (learning phase).

#### 2.3.1 Elevated plus-maze test (EPM)

EPM was used to assess anxiety-like behaviors [22]. The maze consisted of two closed arms (50 cm) and two open arms (50 cm) arranged in a way that identical arms were placed opposite to each other and extended from a central platform (10 × 10 cm). The apparatus was elevated to a height of 60 cm from the floor. During the trials, each rat was placed at the center of the maze facing one of the open arms and allowed to explore the maze for 5 min. During the test, the percent of time and number of entries into the arms was recorded.

#### 2.3.2 Open field test

Open field test was used to measure motor activity and anxiety. All of the animal's behaviours were recorded using Ethovision Software (Noldus Technology, Netherlands). The apparatus consisted of a square arena (90 × 90 × 45 cm) made of Plexiglas. The maze floor was divided by lines into 16 squares that allowed the definition of central and peripheral areas. Each rat was placed in the center of the arena and the behavioral parameters including total distance moved (cm), mobility, total spent in center zone (s), velocity and the number of rearing (animals standing upright on both hind legs in a vertical position) and grooming (The rats lick every reachable parts of their body for maintaining skin cleanliness) were recorded for 5 min. At the end of each session, rats were removed from the maze and the chamber was thoroughly cleaned with a damp cloth and dried. The procedure was performed in a noise and light controlled room [23].

#### 2.3.3 Novel object recognition test (NOR)

Object recognition memory was assessed using a NOR test. The experimental arena consists of a square wooden box with the following measurements: 60 × 60 × 40 cm. This test consisted of three phases: 1) Habituation phase; each animal was exposed to the arena (for a period of 10 min) with no objects and allowed to explore the arena freely for habituation. 2) Training phase; 24h after habituation phase, animals were reintroduced to the arena center exposed to two identical objects that were located in two different corners for a period of 5 min. 3) Test phase; after 45 min, the rats were replaced in the arena which contained one familiar and one novel object for a period of 3 min. Discrimination ratio (as recognition index) was defined as ratio of time spent for exploring each object to the total time spent for exploring both the objects in the training and test phases [24].

#### 2.3.4 Passive avoidance test

A shuttle-box apparatus was used for passive avoidance test, which was composed of two adjacent chambers of identical sizes separated by a guillotine door (light and dark parts) [25]. This test was performed for each rat in two consecutive learning phases at the first day. In the habituation phase, the rats were placed in the light chamber and permitted to enter the dark camber freely to habituate. During the acquisition phase (two hours after the habituation), each rat was located in the light chamber and the guillotine door between the two compartments was opened. Upon the entrance into the dark chamber, the door was closed and an electric foot-shock (0.5 mA, 50 Hz, 2 s once) was delivered through grid floor. After 20 s, the rat was returned to its home cage and 30 min later, the same course was repeated. If the animals did not enter into the dark room, the acquisition of passive avoidance learning was considered successful. Otherwise, the rats received the shock a gain and the number of acquisition sessions was recorded. Twenty-four hours after the learning phase, step-through latency (STL) was recorded up to a maximum of 300 s.

### 2.4 Statistical analysis

GraphPad Prism 8 was used for statistical analysis of data and figure production. Kolmogorov-Smirnov test was used for normality evaluation of all data. Normally distributed data analyzed using a one-way ANOVA test. Where a main effect was seen in ANOVA tests, pairwise comparisons between groups were then made using Tukey's *post-hoc* tests. Results that were not normally distributed, analyzed using a Kruskal-Wallis test. In each case, p < 0.05 was considered statistically significant and all data were expressed as median and interquartile range.

## 3 Results

**Figure 1:**
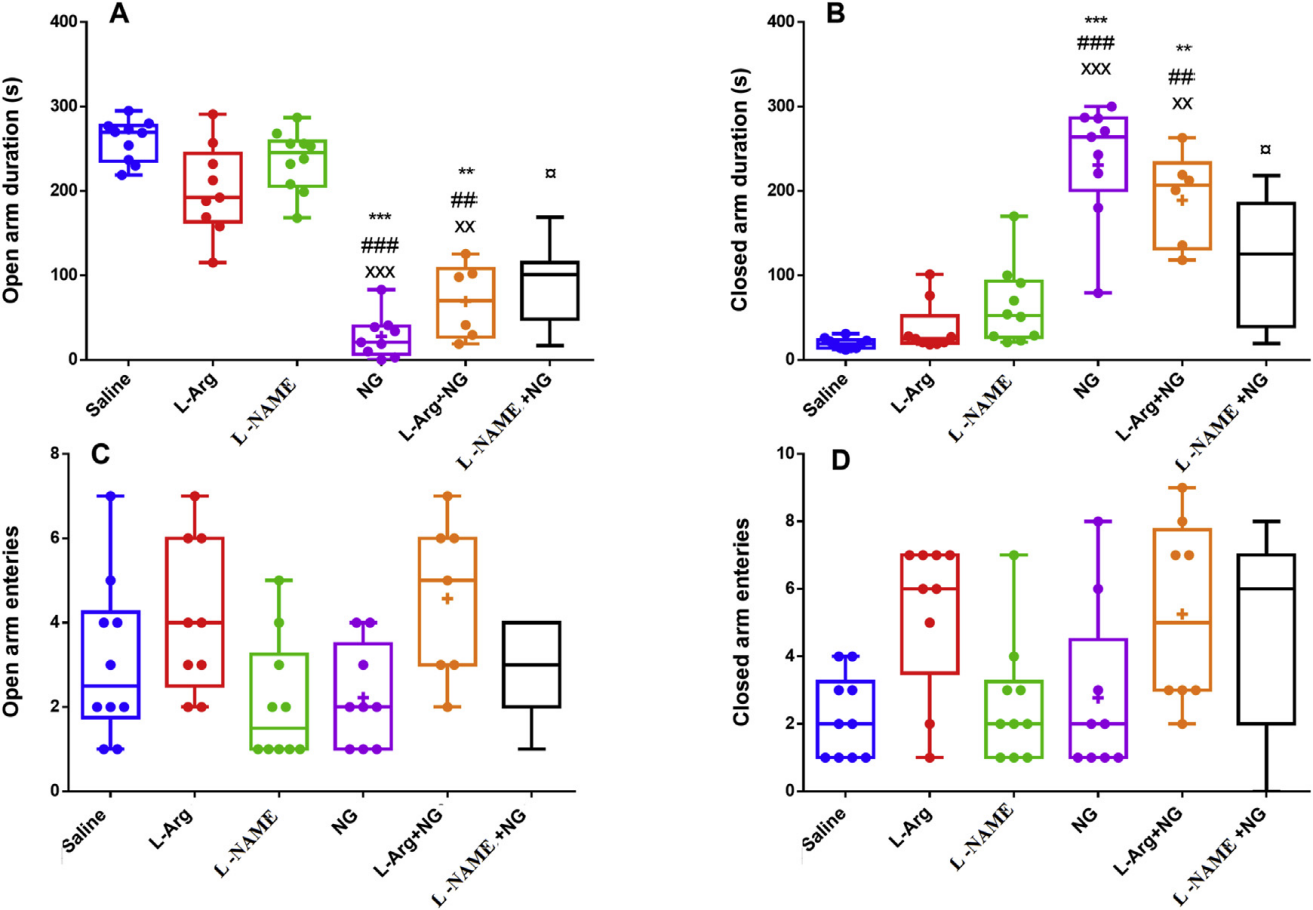
The effect of nitric oxide system on time spent in the open arms (A), closed arms (B) and number of entries to open (C) and closed arms (D) in the EPM. Data presented as median and interquartile range. ∗∗, ∗∗∗: p < 0.01 and p < 0.001 versus saline group; ^##, ###^p < 0.01 and p < 0.001 versus L-Arginine-treated group; ^**××,×××:**^p < 0.01 and p < 0.001 versus L-NAME-treated group and^**¤**^:p < 0.05 versus NTG-treated group.

When anxiety-like behavior was examined using EPM, a main effect of treatment was observed. Administration of NTG (p < 0.001; Figure 1A) and L-Arginine + NTG (p < 0.01) significantly decreased the duration of time spent in the open arms as compared to the animals receiving saline, L-Arginine and L-NAME, while L-NAME treatment (p < 0.05) was able to partly ameliorate NTG effects upon this parameter. Moreover, a main effect of treatment upon closed arm duration was also detected. *Post hoc* pairwise comparisons revealed that closed arm duration was significantly increased by both NTG only and NTG plus L-Arginine treatments (p < 0.001 and p < 0.01; Figure 1B) while L-NAME treatment (p < 0.05) was able to modify NTG effects (p < 0.05; Figure 1B). It means that L-NAME likely attenuated anxiety-like behaviour induction in migraine group. As shown in Figure 1 C and D, there was no significant difference in the numbers of entries in the open and closed arms among experimental groups.

In the locomotor analysis test (Table 1), a overall effect of treatment upon mobility duration, total distance moved and time spent in center zone was detected. As shown in Table 1, these parameters significantly decreased in NTG treated-rats as compared to the saline, L-Arginine and L-NAME groups (p < 0.05). In addition, injection of L-Arginine plus NTG caused a significant reduction in mobility duration and time spent in center zone as compared with the saline, L-Arginine and L-NAME groups (p < 0.05). However, the administration of NTG plus L-NAME significantly suppressed the observed effects of NTG on mobility duration factor and spending time in center zone (p < 0.05). Treatment with NTG plus L-NAME caused to more mobility duration and time spent in the central zone as compared to rats receiving NTG and NTG plus L-Arginine indicated L-NAME injection inhibits the effects of NTG on these parameters.

**Table 1:** The effect of nitric oxide system on locomotor activity in the open field test.

	Saline	L-Arg	L-NAME	NG	L-Arg + NG	L-NAME + NG
Rearing number	5 ± 1	4.2 ± 1	4.3 ± 1	7.6 ± 1.7	5 ± 1.5	2.6 ± 0.7
Grooming number	2.2 ± 0.3	2.4 ± 0.3	2.3 ± 0.7	1.8 ± 0.6	1.6 ± 0.6	2.4 ± 0.2
Mobility duration (s)	0.5 ± 0.1	0.4 ± 0.1	0.49 ± 0.1	0.03 ± 0.0∗^#**×**^	0.05 ± 0.0∗^#**×**^	0.21 ± 0.1^**¤**^
Total distance moved (cm)	2008.1 ± 223	1959.8 ± 184	1926.1 ± 220	1050.8 ± 88.1∗^#**×**^	1512.5 ± 223.2	1488.4 ± 155
Velocity (cm/s)	6.7 ± 0.7	6.5 ± o.6	6.7 ± 0.5	5.8 ± 0.6	6.5 ± 0.8	4.9 ± 0.5
Time spent in center zone (s)	7.8 ± 1.6	6.2 ± 1.3	7.1 ± 1.5	2.3 ± 0.6∗^#**×**^	1.8 ± 0.5∗^#**×**^	6.2 ± 1.9^**¤**^

Data presented as Mean ± SEM. ∗: p < 0.05 versus saline group; ^#:^p < 0.05 versus L-Arginine-treated group;^**×**:^p < 0.05 versus L-NAME-treated group and ^**¤**^:p < 0.05 versusNTG-treated group.

**Figure 2:**
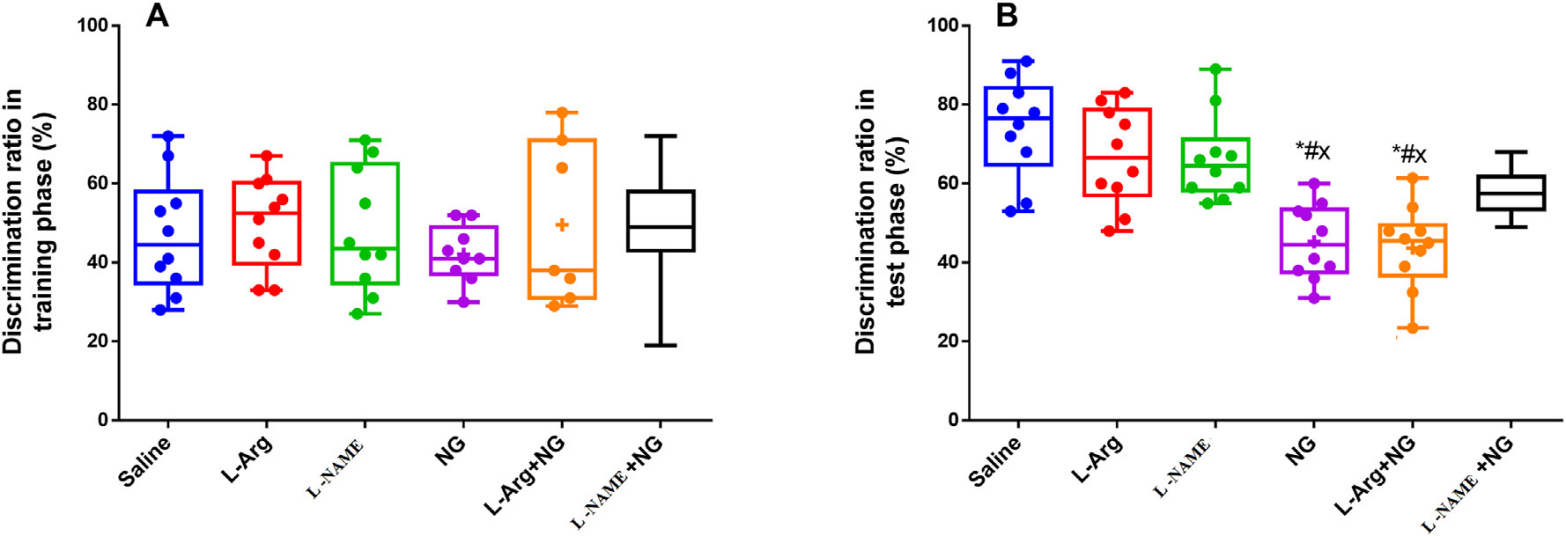
The effect of nitric oxide system on A) discrimination index in training phase and B) discrimination index in retention session in NOR test. ∗: p < 0.05 versus saline group; ^#:^p < 0.05 versus L-Arginine-treated group;^×:^p < 0.05 versus L-NAME-treated group and^**¤**^:p < 0.05 versus NTG-treated group.

In the NOR test, in training phase, all groups spent a similar amount of time exploring the objects and there was no significant difference in the discrimination index between them (Figure 2A). As shown in Figure 2B, NTG and NTG plus L- Arginine treated-rats showed significant reduction of discrimination index for the novel object in retention session as compared to animals receiving saline, L-Arginine and L-NAME (p < 0.05). These data suggest the improvement role of L-NAME in familiar object recognition memory impairment in rats with migraine pain.

**Figure 3:**
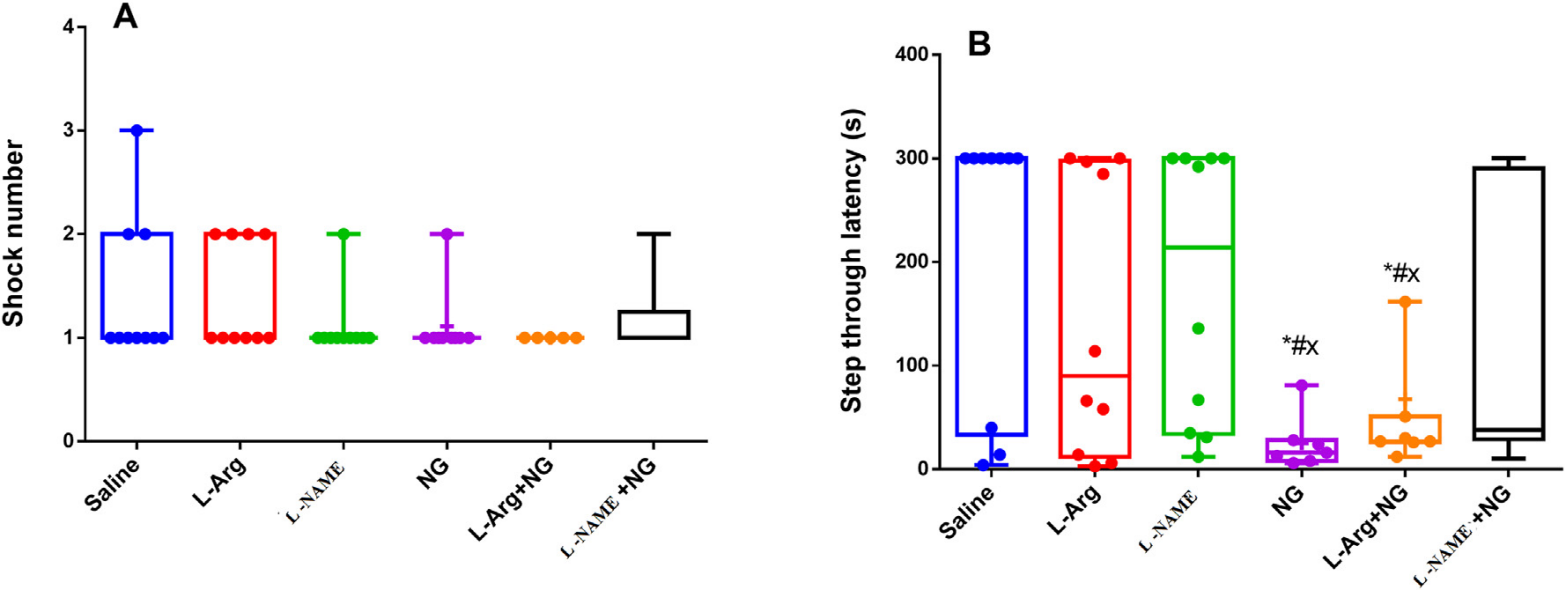
The effect of nitric oxide system on A) shock number and B) step through latency (STL) in shuttle-box test. Decreased STL indicates an impaired fear memory in NTG groups and administration of L-NAME counteracted this impairment. ∗, ^#,×:^p < 0.05 versus saline, L-Arginine and L-NAME-treated groups respectively.

In the passive avoidance test, there was no significant difference among groups in the number of shocks in the acquisition phase (Figure 3A). In the retrieval assessment phase of the test which was undertaken 24 h after learning, an overall effect of treatment upon step through latency was found where post hoc pairwise comparisons revealed that this measure was significantly decreased in both NTG only and NTG plus L-Arginine groups (both p < 0.05, Figure 3B) although the L-NAME plus NTG group was not significantly meaningful when compared with the saline group indicating that L-NAME treatment was partially able to improve the reduction in step through latency induced by NTG.

## 4 Discussion

Migraine is a debilitating chronic condition with cognitive sequences that significantly reduce the quality of life in the patients affected [26]. Animal models of migraine might provide an opportunity for the study of migraine and the plausible mechanisms [27, 28, 29]. Though previous studies have demonstrated cognitive impairments in migraine subjects [10], its neurobiological mechanisms are not clear yet. The present study was conducted to evaluate the effect of chronic migraine-like pain in the animals on cognitive and anxiety-like behaviors and the role of NO pathway was evaluated by administration of NOS inhibitor and NO precursor. Results of this study demonstrated cognitive impairments and increase in anxiety- like behavior in animal model of migraine and NO pathway seems to modulate these alterations.

Dilekoz et al. with Morris water maze test showed spatial memory impairment in FHM1 (familial hemiplegic migraine 1) mutant mice [30]. Results of the current study were in line with their findings so that chronic administration of NTG as an animal model of migraine led to impairments in the spatial and object memory of the animals and NO pathway modulated these effects.

NTG-induced migraine like pain led to an increased anxiety and reduction of locomotor activity in rats. L-NAME attenuated the anxiogenic effects of NTG and reversed the diminished locomotion to some extent. In addition, blockage of NO system reversed the detrimental effects of NTG on spatial learning and memory. Our findings support earlier work showing that chronic pain induced deficits in learning and memory in patients [16]. Structural and functional changes in the hippocampus also were observed in patients with chronic pain [31, 32]. In line with our findings, Maleki et al., reported decrease in hippocampal volume in patients with recurrent migraine attacks [33]. Previous studies had shown the effect of NO on pre and post synaptic pathways involved in long-term potentiation, which is involved in memory [4, 5]. In another study, swimming stress impaired passive avoidance learning, which was ameliorated by L-NAME, while pretreatment with L-Arg had no effect on that [34].

The activation of trigeminal sensory neurons led to transmit nociceptive information into the brainstem and also promote inflammation by releasing of CGRP. The secretion of CGRP activates glial cells that results in increased in expression of iNOS and NO production [3]. In addition, it has been reported that CGRP and NOS co-localize in a significant proportion of trigeminal ganglion neurons [35]. It has been hypothesized that there is an interaction between CGRP and NO in trigeminovascular system and they can amplify each other's activity [36], thus the findings of the current study which demonstrate that cognitive impairments induced by NTG are modulated by NO pathway might be due to the neural changes at the trigeminal ganglion. Future studies are needed to study the effect of local NOS manipulation on NTG-induced cognitive impairments in animal model of migraine.

Our results demonstrated that a moderate, systemic NTG dose migraine elicits an anxiogenic response and decreased locomotion in the animals. Systemic NO antagonist reversed anxiogenic effects of NTG in this model, anxiety like behavior and motor impairments were also partially reversed.

Our findings are in line with previous works on the involvement of the NO system in the modulation of anxiety. Previous studies indicated that NO is localized in regions involved in anxiety behaviors and play a significant role in mediating anxiety behaviors [37]. NOS inhibitors have been reported to show anxiolytic activity in animal models of anxiety-related disorders [38]. Faria et al. (1997) demonstrated that L-NAME administration in the rats increases time spent on open arms, which points to an anxiolytic effect for L-NAME [39]. It has been demonstrated that anxiolytic effects of benzodiazepines are mediated through nitric oxide system as well [40]. A study had also indicated anxiolytic effect of NO in rats that have received L-Arg plus morphine prior to restraint stress [41]. On the other hand, elevated plus maze and forced swim test were performed by Spiacci Jr. et al. (2008) for evaluating the role of NO system in anxiety like behaviors. Behaviors of locomotion and anxiety-like responses were modulated by the nitric oxide system. Different effects on anxiety-like behaviors have seen with administration of different dosages of L-NAME and L-Arginine, so that higher dosage of L-NAME had an anxiogenic effect while low doses demonstrated an anxiolytic effect [15].

Contrary to our results obtained in this model, a single injection of nitroglycerin (NTG) in rats enhanced locomotor activity in comparison to control animals while repeated NTG administration leads to a reduction in locomotion compared to control group (for review see Vuralli et al., 2019) [42]. In another study, Nasehi et al. (2012) using non-cholestatic mice indicated that pretreatment with several doses of L-arginine increased the number of head-dips, no change in locomotor activity and head dip latency was observed, which represents an anxiolytic effect for L-Arginine. They also reported that pretreatment with several doses of L-NAME attenuated the number of head dips in non-cholestatic mice, no change in locomotor activity and head dip latency was observed, which represents an anxiogenic effect of L-Arginine [43]. Dual effects following intervention with NO-mediated neurotransmission have been widely reported in several behavioral tests [44, 45], including animal models of anxiety and depression which demonstrates that NO system modulates anxiety-like behaviors and locomotion, while the dosage of pharmacologic agents and time of administration significantly alter the direction of these changes.

Passive avoidance learning and object recognition memory were impaired following administration of NTG in rats and inhibition of the NO pathway reversed this effect, While L-Arginine enhanced deficits in passive avoidance learning and object recognition memory. Deficits in learning and memory and increased anxiety and depression has been reported in chronic pain patients [45, 46]. An increased impairments in cognitive dysfunctions and psychological symptoms has been reported in migraine patients [10, 11]. It has been demonstrated that migraine patients have deficits in cognitive performance and attention than non-migraines. Increased frequency and the longer durations of migraine attacks are associated with more severe subjective cognitive decline [10, 11].

In the central nervous system, NO as a neurotransmitter modulates various neuronal functions. NO can act as an important mediator in the regulation of excitability and firing and several forms of memory formation. In addition, it has been shown that this messenger is implicated in synaptic plasticity, LTP and the consolidation of LTP [47]. Inhibition of NO production has been shown to impair the consolidation of memory and block the induction of LTP [48]. It has also been demonstrated that L-arginine, an NO precursor, improves memory development and inverts the effect of NOS inhibition in the animals [49]. Chronic administration of L-Arginine has been shown to impair spatial learning and memory [50]. Other researchers evaluated the effects of acute administration of L-arginine on learning and memory, but in our study the rats were chronically treated on alternate day for 9 days and addition of L-Arginine to NTG led to a more pronounced cognitive function in the animal model of migraine.

One of the limitations to the current study is the lack of behavioral assays for migraine like pain in the animals. But previous studies have recruited chronic NTG as an animal model of migraine and demonstrated that it could mimic clinical migraine pain in the animals.

In conclusion, based on an NTG-induced migraine model, it was found that L-arginine/NO pathway can disturb learning and memory and elicit anxiety like behavior in rats. In addition, it seems that the inhibition of nitric oxide system through L-NAME administration partially reverses these impairments. Future studies might evaluate the effect of localized NO system inhibition in brain regions corresponding for migraine pain such as trigeminal ganglion neurons on cognitive impairments in animal model of migraine. Furthermore, considering the profound impact of sex hormones on migraine pain, future studies might recruit procedures such as castration and ovariectomy to evaluate the role of sex hormones on cognitive impairments in animal model of migraine.

## Declarations

### Author contribution statement

Parichehr Taheri: Conceived and designed the experiments; Performed the experiments; Analyzed and interpreted the data; Wrote the paper.

Fatemeh Mohammadi: Conceived and designed the experiments; Performed the experiments; Contributed reagents, materials, analysis tools or data.

Masoud Nazeri, Mohammad Reza Zarei, Goli Charmani, Mohsen Abedini Esfahlani, Farahnaz Taheri: Analyzed and interpreted the data; Wrote the paper.

Mohammad Shabani: Conceived and designed the experiments; Analyzed and interpreted the data.

### Funding statement

Parichehr Taheri was supported by 10.13039/501100004621Kerman University of Medical Sciences (Grant for the DDS thesis).

### Data availability statement

Data will be made available on request.

### Declaration of interests statement

The authors declare no conflict of interest.

### Additional information

No additional information is available for this paper.
